# Regional effect on the molecular clock rate of protein evolution in Eutherian and Metatherian genomes

**DOI:** 10.1186/s12862-021-01882-x

**Published:** 2021-08-04

**Authors:** Raf Huttener, Lieven Thorrez, Thomas in‘t Veld, Barney Potter, Guy Baele, Mikaela Granvik, Leentje Van Lommel, Frans Schuit

**Affiliations:** 1 Gene Expression Unit, Dept. of Cellular and Molecular Medicine, KU Leuven, Herestraat 49, O&N1, Bus 901, 3000 Leuven, Belgium; 2 Tissue Engineering Laboratory, Department of Development and Regeneration, KU Leuven, Kortrijk, Belgium; 3 Department of Microbiology, Immunology and Transplantation, Rega Institute, KU Leuven, Leuven, Belgium

**Keywords:** Landscapes, Protein evolution, GC content, Metatheria, Eutheria, Molecular clock, Substitution rate, Neutral evolution

## Abstract

**Background:**

Different types of proteins diverge at vastly different rates. Moreover, the same type of protein has been observed to evolve with different rates in different phylogenetic lineages. In the present study we measured the rates of protein evolution in Eutheria (placental mammals) and Metatheria (marsupials) on a genome-wide basis and we propose that the gene position in the genome landscape has an important influence on the rate of protein divergence.

**Results:**

We analyzed a protein-encoding gene set (n = 15,727) common to 16 mammals (12 Eutheria and 4 Metatheria). Using sliding windows that averaged regional effects of protein divergence we constructed landscapes in which strong and lineage-specific regional effects were seen on the molecular clock rate of protein divergence. Within each lineage, the relatively high rates were preferentially found in subtelomeric chromosomal regions. Such regions were observed to contain important and well-studied loci for fetal growth, uterine function and the generation of diversity in the adaptive repertoire of immunoglobulins.

**Conclusions:**

A genome landscape approach visualizes lineage-specific regional differences between Eutherian and Metatherian rates of protein evolution. This phenomenon of chromosomal position is a new element that explains at least part of the lineage-specific effects and differences between proteins on the molecular clock rates.

**Supplementary Information:**

The online version contains supplementary material available at 10.1186/s12862-021-01882-x.

## Background

Recently we proposed a method that facilitates genome-wide analysis of macro-evolutionary events in vertebrates [1]. We used a sliding window approach, which integrates information of a centered gene and its 100 neighbors, smoothening the known erratic behavior of individual genes that vary greatly in nucleotide composition, intron size and rate of evolution of encoded proteins [1]. Such integration visualizes strong region-specific events that apply to tens or hundreds of adjacent genes. This enables the calculation of chromosomal heterogeneity, which can be visualized as landscapes of base composition of encoded mRNAs, amino acid composition of encoded proteins and rates of protein evolution [1]. A previous analysis of 55 genomes—including species of all major vertebrate classes—resulted in landscapes with conserved regions in which GC content (GC%) of encoded mRNAs, abundance of glycine, alanine, arginine and proline (GARP%) in encoded proteins and divergence rates of orthologous proteins were clearly below or above genome-wide averages. In particular, regions near telomeres exhibited elevated GC% and GARP% in the class of mammals [1].

In this study we addressed whether the analysis of genome-wide landscapes of protein divergence in different mammalian species could shed new light on the discussion which factors contribute to the molecular clock rate of protein evolution. Initial studies by Zuckerkandl and Pauling suggested a constant rate k in various organisms at which amino acid changes accumulate in proteins such as cytochrome c, hemoglobin and fibrinogen [2, 3]. This constant rate inferred the existence of a species-independent molecular clock constant of orthologous proteins. However, one complexity of the concept of a molecular clock is the fact that k varies enormously between different types of proteins. Moreover, it was observed that for the same type of protein, the rate k can vary between lineages, so that data were better explained by relaxed clock models [3, 4]. Different rates for different species were proposed to depend on multiple factors such as generation time, population size, and basal metabolic rate [5]. To the best of our knowledge, chromosomal positioning of a gene (which often differs in different lineages) has not yet been considered as an underlying mechanism. To this end, we analyzed two major lineages of mammals that split approximately 160 million years ago [6, 7] into *Eutheria* (placental mammals) and *Metatheria* (marsupials). While genome-wide averages of protein divergence suggest the existence of a clock rate, these averages are made up of individual protein data with enormous differences in rates. However, when we take samples of 101 genes based on the location in the genome, landscapes with increased and decreased rates can be discerned. As the Metatherian and Eutherian landscapes display different areas of deceleration/acceleration, we propose that gene position is a mechanism that can contribute to differences between phyla in the rate of orthologous protein evolution.

## Results

**Fig. 1:**
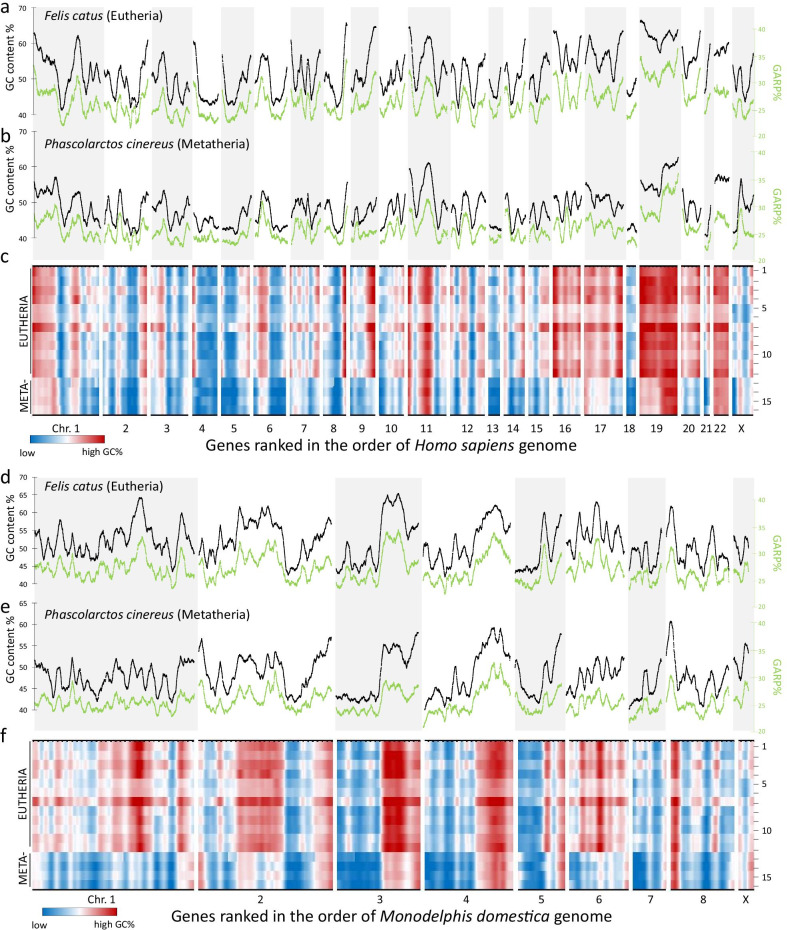
Landscapes of GC% and GARP% in Eutheria and Metatheria. For each of the 15,727 genes included in this comparative analysis we calculated GC content (GC%) and relative occurrence of glycine, alanine, arginine and proline in predicted proteins (GARP%). Gene data were integrated with the values of surrounding genes (50 genes on each side). Alternating grey and white zones demarcate individual chromosomes. In **a–c**, genes were ordered according to a Eutherian (*Homo sapiens*) genome, while in **d–f** the gene order was Metatherian (*Monodelphis domestica*). a-b) GC% (black) and GARP% (green) landscape distribution for a Eutherian (*Felis catus*; cat, **a**) and a Metatherian (*Phascolarctos cinereus*; koala, **b**). **c** A heatmap of the GC% landscape for all 12 Eutheria and 4 Metatherian in the following order: 1 *Sus scrofa*, 2 *Equus asinus*, 3 *Orcinus orca*, 4 *Felis catus domestica*, 5 *Cricetulus griseus*, 6 *Rattus norvegicus*, 7 *Oryctolagus cuniculus*, 8 *Callithrix jacchus*, 9 *Trichechus manatus latirostris*, 10 *Loxodonta africana*, 11 *Chrysochloris asiatica*, 12 *Orycteropus afer afer*, 13 *Vombatus ursinus*, 14 *Sarcophilus harrisii*, *15 Phascolarctos cinereus*, 16 *Monodelphis domestica*. In the gene ranking of both genomes a distinct Eutherian and Metatherian GC% landscape can be discerned

**Fig. 2:**
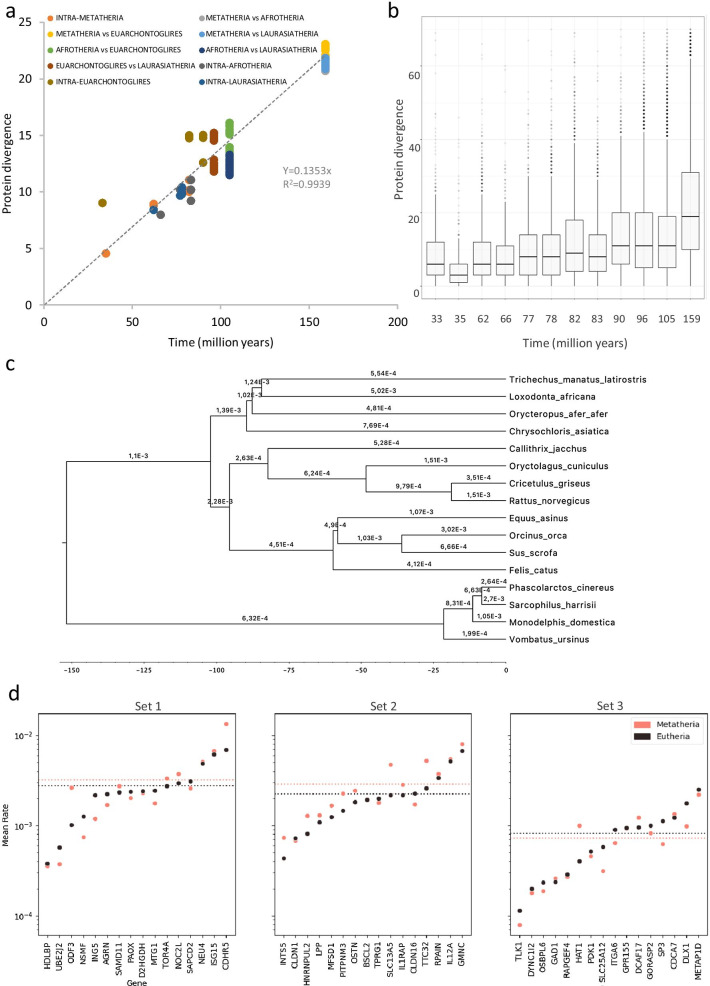
Evolutionary rates based on observed protein divergence (**a**, **b**) and phylogenetic analysis (**c**, **d**). **a** For each pairwise species comparison (total of 120 comparisons), divergence time (obtained from timetree.org) versus average protein divergence was plotted. Average protein divergence was the average divergence of all orthologous proteins. Data points are colored according to clade comparisons as indicated in the legend. The best regression line (dashed) was fitted to the data; **b** Boxplots of individual protein divergences; **c** Proof-of-concept phylogenetic analysis (detailed in the methods section) for the NSMF gene, with branches annotated with their inferred evolutionary rate (in substitutions per site per million years); **d** Mean evolutionary rates for Eutheria (black) and Metatheria (red) estimated through phylogenetic analysis, conducted on three sets of 16 genes with complete protein sequence information available for all species. Set 1: Eutherian subtelomeric genes located in landscape windows with highest protein divergence rates in eutherian comparisons; set 2: Metatherian subtelomeric genes in windows with highest protein divergence rates for intra-Metatherian comparisons; set 3: genes that were not in subtelomeric of Eutherian and Metatherian genomes with low protein divergence rates for intra-Metatherian and intra-Eutherian comparisons

The present work departed from methods that were described recently to characterize protein-encoding genes of vertebrate genomes [1]. For this approach, homologous genes of different species were ranked on a reference genome and parameters associated to the genes were plotted, giving rise to exome landscapes, which allow comparisons between multiple genomes. In the current study, we compared these landscapes between two major classes of mammals: *Eutheria* (12 species) and *Metatheria* (4 species). Figure 1a and b illustrate the exome landscape characteristics of the sliding window average of GC content (GC%) and the sum of glycine, alanine, arginine and proline in the amino acid composition (GARP%) of two mammals that are comparable in terms of body size and life span: *Felis catus* (cat, eutherian lineage, Fig. 1a) and *Phascolarctos cinereus* (koala, metatherian lineage, Fig. 1b). When analyzed per species, the correlation between GC% and GARP% was very high (R = 0.94 for koala and R = 0.92 for cat). However, inter-species correlations were much lower: R = 0.78 for GC% and R = 0.82 for GARP%. Figure 1d and 1e show the same data set, but with the difference that the genes were ordered according to the metatherian *Monodelphis domestica* reference genome. Again, within species, the correlation between GC% and GARP% was excellent (R > 0.92), while inter-species correlations for GC% (R = 0.75) and GARP% (R = 0.80) were lower. While Fig. 1a, b, d and e illustrate the myriad details in the genome landscapes of two species, they do not allow for a practical search for lineage-specific events involving multiple species. Yet, such an analysis is useful as lineage-specific details in the landscapes could be taken as synapomorphies to further study genome evolution. For all of the 16 studied species we calculated the sliding window averaged GC% values, creating landscapes that can visualize regional differences of low (blue) and high (red) GC% (Fig. 1c and f). Whether gene regions are calculated using Eutherian reference genomes (*Homo sapiens*—Fig. 1c or *Sus scrofa*—Additional file 1: Fig. S1) or a Metatherian reference genome (*Monodelphis domestica*—Fig. 1f), the strong lineage-specific characteristics of the generated landscapes were clear and prominent. Indeed, within the Eutherian group only minimal differences were observed between Laurasiatheria (line 1–4), Euarchontoglires (line 5–8) and Afrotheria (line 9–12), despite large morphological differences (life history traits) between individual species (*e.g*. rat (line 6) versus elephant (line 10)). Moreover, the four Metatherian GC% landscapes were highly similar, irrespective of the chosen reference genome. In contrast, for each of the reference genomes, landscapes were clearly different between the Eutherian and Metatherian lineages (line 1–12 vs. line 13–16). Most of the areas with peak GC% levels in one lineage only were located in subtelomeric regions when evaluated using the reference genome of the lineage. Examples of Eutherian-specific subtelomeres with high GC% in the human gene order are the subtelomeres of the p-arms in chromosomes 4, 16 and 19, and the subtelomeres of the q-arms of chromosomes 5, 7, 8, 9, 14, 16, 17 and X. When orthologous genes were ordered based on position of another Eutherian reference genome (*Sus scrofa*), the highest GC% was also subtelomeric despite the different karyotype (Additional file 1: Fig. S1). However, when genes were ranked in the order of the Metatherian *Monodelphis domestica* genome (Fig. 1f), most of the Eutherian peak levels of GC% were far from telomeres. Instead, Metatherian-specific peak values of GC% were observed at the subtelomere of the p-arm of chromosome 2 and the subtelomeres of the q-arm of chromosomes 1, 6 and X. Noteworthy is the common Eutherian/Metatherian GC% enrichment on  the subtelomere of the p-arm of human chromosome 11. Next, we assessed whether subtelomeric GC-rich regions with an elevated contribution of GARP% to the amino acid composition of the encoded proteins could coincide with regions where proteins underwent accelerated evolution. In a first step, we calculated for all proteins and all species the pairwise protein divergence. Sixteen species make for 120 pairwise comparisons: 1–66 intra-Eutherian, 67–114 Eutherian-Metatherian, 115–120 intra-Metatherian. For each pairwise comparison, based on all orthologous protein divergences, the average protein divergence (PD^av^%) was calculated. We then compared the relationship between the time to the last common ancestor (t) versus PD^av^% (Fig. 2a). In a neutral model of evolution with a strict clock constant and without saturation, all data would fit to a line that originates in the X/Y intersection: PD^av^% = k^av^ • t. We observe an almost perfect linear relationship with a good fit of the data to the regression line (R^2^ > 0.99), suggesting an average genome-wide molecular clock constant of 1.3% protein divergence per 10 million years of evolution (Fig. 2a). Moreover, it was noticed that the Eutherian-Metatherian comparisons (t = 160 million years) fit perfectly to this line. It is well known however, that these genome-wide averages are based on individual proteins with vastly different rates of evolution: between genes in one organism and between species for one orthologous gene. Indeed, this is also clear in our data set (Fig. 2b) where the distribution of divergence of individual proteins is shown in a box plot display. Major differences in the evolutionary rates of protein divergence were also noted in a proof-of-concept phylogenetic analysis conducted on three sets of 16 genes with complete protein sequence information available for all 16 studied species: set 1 (n = 16), Eutherian subtelomeric genes located in landscape windows with highest protein divergence rates in eutherian comparisons; set 2 (n = 16), Metatherian subtelomeric genes in windows with highest protein divergence rates for intra-Metatherian comparisons; set 3 (n = 16), genes that were not in subtelomeric regions of Eutherian and Metatherian genomes with low protein divergence rates for intra-Metatherian and intra-Eutherian comparisons. An example of phylogenetic data is shown for the NSMF gene (set 1—Fig. 2c): different branches of the inferred evolutionary tree have different rates of protein divergence, so that data fitted best to a relaxed clock model. Similar data were observed for most of the studied genes from the three sets. The estimated rates (expressed in a logarithmic scale on the Y-axis of Fig. 2d) disclose a heterogeneous picture in both clades with up to two orders of magnitude in the variation of protein divergence rates between individual genes. Genes with a higher evolutionary rate in Eutheria are slightly more common in set 1, while genes with a higher evolutionary rate in Metatheria are enriched in set 2. As expected from the landscapes, the average rate for genes from set 3 is one order of magnitude lower than groups 1 and 2 (Fig. 2d). Taken together, phylogenetic analysis confirms the idea that a genome-wide molecular clock constant is based on averaging genes with vastly different rates of evolution, often with significant variation between species. Such phylogenetic analyses come at a considerable computational cost however, owing to the complexity of performing model comparisons under these clock models and to analysing amino acid data sets using high-dimensional models.

We were interested in the possibility that the regional position of a gene could influence rates between individual protein types and could contribute to differences between species for orthologous proteins. To assess this possibility, the protein divergence was normalized to observe deviations from the genome average:

$${\text{nPD}}\% \, = \,{{{\text{PD}}\% } \mathord{\left/ {\vphantom {{{\text{PD}}\% } {{\text{PD}}^{{{\text{av}}}} \% }}} \right. \kern-\nulldelimiterspace} {{\text{PD}}^{{{\text{av}}}} \% }} \, \, = \,{{\left( {{\text{k}}.{\text{ t}}} \right)} \mathord{\left/ {\vphantom {{\left( {{\text{k}}.{\text{ t}}} \right)} {\left( {{\text{k}}^{{{\text{av}}}} \cdot {\text{t}}} \right)}}} \right. \kern-\nulldelimiterspace} {\left( {{\text{k}}^{{{\text{av}}}} \cdot {\text{t}}} \right)}} \, \, = \,{k \mathord{\left/ {\vphantom {k {{\text{k}}^{{{\text{av}}}} }}} \right. \kern-\nulldelimiterspace} {{\text{k}}^{{{\text{av}}}} }}$$

**Fig. 3:**
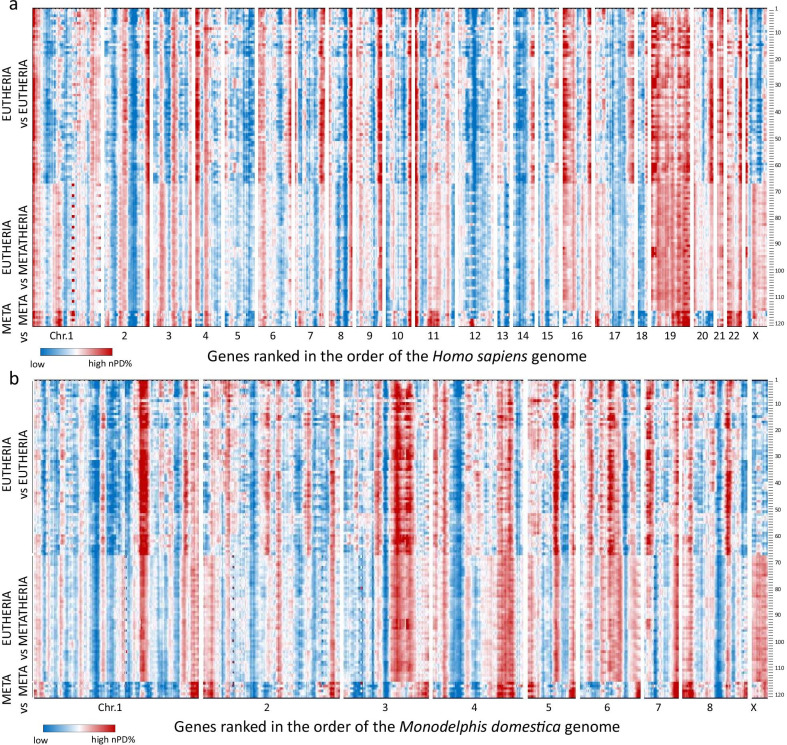
Regional changes in the molecular clock rates of orthologous proteins in Eutherian and Metatherian genomes. The heatmaps with a human (**a**) or *Monodelphis domestica* (**b**) ranking order each integrate approximately 1.8 million pairwise comparisons of orthologous proteins departing from the core set of 15,727 mammalian proteins and 120 pairwise comparisons between 16 species. For each comparison, the %protein divergence (PD% = 100—%identical amino acids) was divided by the average protein divergence between the same two species. The resulting normalized %protein divergence (nPD%) represents **k/k**^**av**^, which is the molecular clock rate of the particular protein divided by the genome-wide average of the molecular clock constant. Sliding window analysis integrates the **k/k**^**av**^ of the centered gene and its 100 neighbors (50 on each side). Details of the 120 comparisons (1–66 intra-Eutherian, 67–114 Eutherian-Metatherian, 115–120 intra-Metatherian). can be found in Additional File 2: Table S1. Most clear-cut landscapes with Eutherian and Metatherian signatures are seen with the intra-Eutherian and intra-Metatherian comparison

It is clear that such normalization eliminates the influence of the phylogenetic distance on the measured result. We therefore aligned (both in the gene order of the human (Fig. 3a) and *Monodelphis domestica* (Fig. 3b) reference genomes) the nPD% landscapes calculated from 120 pairwise comparisons. A detailed list linking each number to a species pair is shown in Additional file 2: Table S1. Each of the heat maps in Fig. 3 is therefore based on about 1.8 million pairwise comparisons of orthologous proteins. Both heat maps clearly display Metatherian and Eutherian signatures of the chromosomal regional position on the relative height of the molecular clock rate. The positions of the regions with the highest nPD% values were often found in subtelomeric regions: on the human reference genome for the intra-Eutherian comparisons (Fig. 3a, line 1–66) and on the *Monodelphis domestica* reference genome for the intra-Metatherian comparisons (Fig. 3b, line 115–120).

**Fig. 4:**
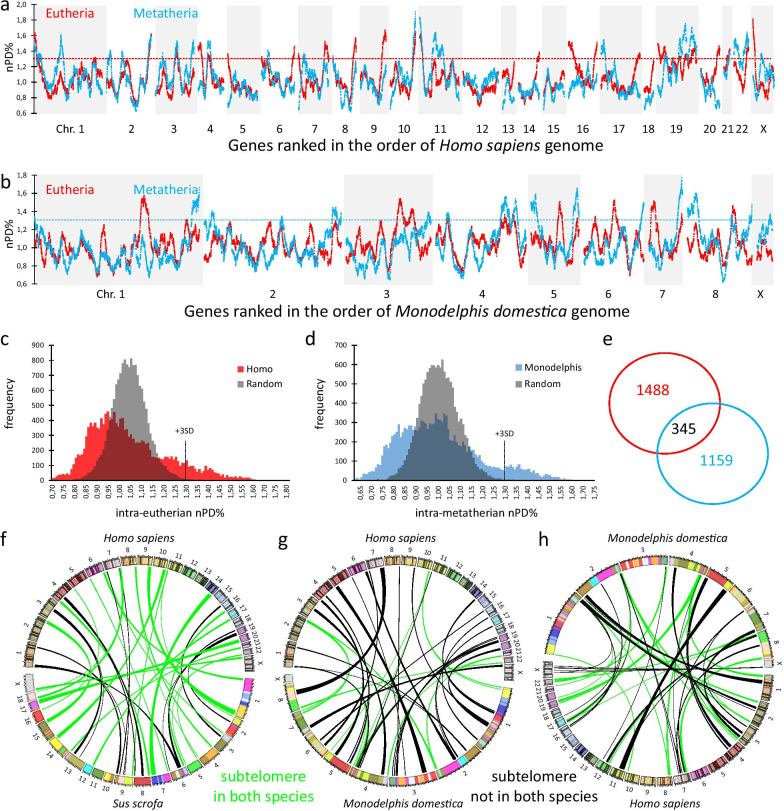
Strong conservation of regional effects in the molecular clock rate in Eutherian and Metatherian landscape signatures. The data set of Fig. 2 was further integrated by calculating the mean sliding window values of 66 intra-Eutherian (red) and nPD% is shown while in blue the average of the 6 intra-Metatherian (blue) comparisons, using the human (**a**) or *Monodelphis domestica* (**b**) genome order. Clear-cut subtelomeric increments above the genome-wide averages (dashed lines) can be observed with discriminative intra-Eutherian and intra-Metatherian maximal values (e.g., the right telomere of human chromosome 14 or *Monodelphis domestica* chromosome 8). Distribution of the window-averaged nPD% is fundamentally different when genes were in random order (grey) as compared to the order of the human genome (red, **c**) or *Monodelphis domestica* genome (blue, **d**). When using a threshold based on mean + 3SD of the random distribution we found 1,833 eutherian windows and 1504 Metatherian windows with window-averaged nPD% exceeding these thresholds. **e** These two gene sets show minimal overlap, providing further evidence for a Eutherian and Metatherian signature of regional effects in the molecular clock rate**.** That subtelomeric regions with high window-averaged nPD% remain subtelomeric despite changes in karyotypes is suggested by a Circos plot (**f**) that connect in two Eutherian species (pig and human) concordant subtelomeric regions with high window-averaged nPD% (green lines) as opposed to few discordant subtelomeric regions with high window-averaged nPD% in one species only (black lines). In contrast human subtelomeric regions with high window-averaged nPD% are predominantly non-subtelomeric in the *Monodelphis domestica* genome (black lines **g**). Similarly, *Monodelphis domestica* subtelomeric regions with high window-averaged nPD% are predominantly non-subtelomeric in the human genome (black lines **h**)

As nPD% is independent of phylogenetic distance, lineage-specific positioning is a major factor for nPD%. We calculated for the 66 different eutherian-eutherian comparisons the mean nPD% for each predicted protein and then constructed a genome-wide landscape based on the sliding window analysis (red lines in Fig. 4a, human reference genome and Fig. 4b *Monodelphis domestica* reference genome). Likewise, we calculated per protein the mean nPD% for the 6 Metatherian-Metatherian comparisons (blue lines in Fig. 4a and b). These mean normalized values clearly show a landscape with regional accelerations (of 30% or more) above the normalized genome-wide means typical for the two mammalian lineages. In order to assess the statistical likelihood for such an outcome and to quantify the number of sliding windows with divergence values above such a threshold, we distributed the 15,727 genes in a random order over the packages that mimic the human or *Monodelphis domestica* chromosomes. We created three random genome orders for the Eutherian and Metatherian analyses and observed a normal distribution of nPD% in both cases. The average of these randomized distributions for Eutheria and Metatheria is shown in Fig. 4c and 4d (grey color). This outcome was fundamentally different from the result of the biologically ordered set of genes in the human order (red distribution, Fig. 4c) or *Monodelphis domestica* order (blue distribution, Fig. 4d). We took a threshold for accelerated divergence using the mean value of the three random sets + 3 standard deviations (SD) (which results in approximately 30% more divergence than the genome-wide mean). These thresholds are shown as dashed lines in Fig. 4c and d. If the genome was completely reshuffled, only 0.3% (n = 47) of the data points were above this threshold. In the actual human order of the genes, however, 1,833 eutherian genes (12% of all studied genes) were observed in regions with divergence rates (of the sliding window) above this threshold, illustrating the strong effect of neighboring genes. The distribution of the sliding window protein divergence in the real gene order is far from a normal distribution and a subpopulation is skewed to high divergence values. The genome location of these windows in a landscape setting can be seen above the red dashed line in Fig. 4a. The same phenomenon was observed when analyzing Metatherian-Metatherian pairwise divergence, studying the genes in a random order or the biological order of the *Monodelphis domestica* reference genome. Again, the number of sliding windows exceeding the 3SD above mean in the three random sets was much smaller (n = 28; 0.6% of all windows) than observed in the analysis of the Metatherian gene order (n = 1,504; location can be seen above the blue dashed line in Fig. 4b). Of interest is the Venn diagram in Fig. 4e which shows only a relatively small overlap between the set of proteins that exhibit both accelerated Eutherian-Eutherian and Metatherian-Metatherian divergent evolution (a list of the 1,833 Eutherian, 1,504 Metatherian and 345 common genes is provided in Additional file 3: Table S2). The positions of the accelerated regions confirm what we already described (Fig. 2), where particular subtelomeres strongly have Eutherian-Eutherian but not Metatherian-Metatherian divergence (human reference gene order) and vice versa (*Monodelphis domestica* gene order).

Defining the first 50 and last 50 genes (windows) of the 23 human chromosomes as subtelomeric, the actual composition of subtelomeric windows in the Eutherian-Eutherian comparisons with divergence exceeding mean + 3SD (908/1,833 = 50%) is greater than randomly expected (2,300/15,757 = 15%). This result is counterintuitive, considering the many chromosome rearrangements such as fusions, fissions and translocations among mammalian species [8]. But it could be explained if subtelomeric regions would stay in a subtelomeric region, even while changing position in genome space during rearrangements. On the contrary, a well-conserved Metatherian karyotype (2n = 14) with a low amount of large chromosomes [9], has enabled a different course with variant choices for subtelomeric accelerations when compared to the eutherian line. We illustrate this with Circos plots connecting the protein-encoding genes between the genomes of *Homo sapiens* and *Sus scrofa* (pig) with accelerations in the Eutherian-Eutherian comparisons (Fig. 4f). In this plot, regions that are subtelomeric in both genomes are colored green showing that these genes remained predominantly subtelomeric in both genomes. The same gene set connecting the genomes of *Homo sapiens* and *Monodelphis domestica* shows predominantly black lines as the loci in the *Monodelphis domestica* genome are not subtelomeric (Fig. 4g). The same was observed for the fast-evolving metatherian genes from the *Monodelphis domestica* genome when these were mapped on the human genome (Fig. 4h). Taken together, Eutherian and Metatherian landscapes—both at the level of GC%, GARP% and protein divergence—are well conserved within each lineage but also contain important differences between lineages, most notably in subtelomeres. Heterogeneity is considerable, so that fluctuation (especially the acceleration of pairwise protein divergence) above the genome-wide average can amount to 30% or more.

## Discussion

We previously described a method to assess large-scale macro-evolutionary events in vertebrate genome-wide landscapes [1]. The current study was focused on the assessment of lineage-specific regional effects of chromosome position on the molecular clock rates of divergent evolution of orthologous proteins. Our analysis has found evidence for these regional effects underlying hallmarks of a Eutherian and a Metatherian direction of genome evolution. The remarkable conservation of exome landscapes among Eutheria and among Metatheria, but clearly differing between those groups, seems to indicate that one key event, 160 million years ago, steered the Metatherian and Eutherian lineages into fundamentally different directions. This key event may be the lineage-specific position of a set of genes that was "externalized" into subtelomeric gene-dense regions. Stochastic redistribution of genes over distinct karyotypes at the time of this split may have defined these two diverging directions. The Circos plot analysis of human and porcine subtelomeres supports the hypothesis that after the split from a common ancestor, a large proportion of the genes that were originally "externalized" remained "externalized" until the present. The implication is that over deep evolutionary time a vast opportunity was created for myriad changes in the amino acid sequence of the encoded proteins. The more rapidly evolving regions are rich in G and C bases and gene-dense. The underlying mechanism behind the GC enrichment is GC-biased gene conversion, which occurs during meiotic recombination [10]. If a mismatch in heteroduplex DNA is present during the recombination event, there is a higher probability of repair towards the G or C allele, which could lead to fixation of the G or C allele in the population. The recombination process has the evolutionary advantage of selection of haplotypes in which epistatic interactions between different alleles of neighboring genes creates novel phenotypes. In this light, the mixture of genes with different functions in one genome locus creates an enormous potential for novel haplotypes that could accelerate the evolution of integrated physiology. Many detailed follow-up studies will be needed to better define the exact impact of gene mutations that contributed to the two diverging evolutionary paths. We realize that this analysis is far from complete. A limitation of our work is the fact that some large gene families were excluded from the study as orthology is elusive because of multiple events of gene gain and loss in different species (e.g., olfactory receptor gene, zinc finger gene clusters, immunoglobulin and T-cell receptor gene clusters). A second point of consideration is that we restricted our analysis to the coding part (exome) of mammalian genomes, while accelerated mutation rates of regulatory, non-coding information could also drive divergent evolution of different lineages. For example in three genes (DIO3, HOXA13, and IGF2), which we will further discuss below because of their remarkable position in the genome landscapes (Additional file 4: Fig. S2a), large CpG islands are not only part of the coding sequence, but also part of a bidirectional promoter region that regulates an opposite strand non-coding RNA (DIO3-AS HOXA13-AS and IGF2-AS—Additional file 4: Fig. S2b). In this context it is remarkable that the 330-nucleotide CpG island on the IGF2 promoter is one of the largest of more than 200 islands in the gene-dense chr11p15.5 subtelomeric region (red circle in Additional file 5: Fig. S3). Genomic imprinting was observed in approximately 1% of the ± 17,000 protein-encoding mammalian genes [11]. Interestingly, this small number of genes is further characterized by an anatomic clustering in a handful of genomic loci that are considered crucial for prenatal growth regulation, development of brain function and postnatal energy homeostasis [11]. It is no coincidence that our approach of mammalian genome landscapes, with massive GC accumulation on a megabase scale, led us to two of these imprinted regions, DIO3 and IGF2, that harbor master genes for the divergent prenatal growth regulation in Metatheria and Eutheria.

Through a systematic study of 15,727 common genes in 16 mammals, we obtained conserved regions of strongly enriched GC% and GARP% in Eutherian and Metatherian landscapes that signify a common mammalian descent. One of the most prominent examples of such a common feature in the two mammalian taxa is the subtelomere of chromosome 11 (chr11p15.5), a 2.5 MB region which is not only very gene dense, harboring ± 200 CpG islands (Additional file 5: Fig. S3) but which also has a much lower GC% in the landscapes of reptilian, amphibian and fish taxa [1]. This common mammalian region with high GC% contains the CDHR5 and ODF3 genes, which were present in set 1 of the phylogenetic analysis, explaining high rates of protein divergence both in Eutheria and Metatheria. Interestingly, the same region contains the IGF2-insulin-tyrosine hydroxylase locus, which encodes key genes for a neuroendocrine network that controls metabolism, nutrition and growth. Indeed, there is widespread consensus about the idea that the mammalian IGF2 locus and the mammalian insulin gene are both crucial for fetal nutrition and growth, balancing the nutritional capacity of the mother to the needs of the developing fetus(es) [12]. Both the maternal adaptation of pancreatic beta cells during pregnancy [13] and the important role of the imprinted IGF2 locus for fetal growth [14] have been studied in detail during the past decades. Human errors in the epigenetic control of this region result in fetal growth disorders such as the Silver-Russell (small fetal size) and Beckwith-Wiedemann (macrosomia) syndromes [12]. Furthermore, abnormal processing of IGF2 was suggested as a mechanism involved in fetoplacental growth restriction [15, 16]. Although the imprinted IFG2 locus and the metabolic importance of insulin is common to Eutheria and Metatheria, we were surprised to note that the IGF2, insulin, tyrosine hydroxylase gene cluster is poorly mapped in some of the marsupial genomes. In fact, for all of these three genes no refseq sequence can be found in the well-studied *Monodelphis domestica* genome according to the public databases (NCBI and Ensembl), something that needs further attention. However, using fluorescent in situ hybridization (FISH), the *M. domestica* IGF2 gene was mapped to the subtelomeric q-arm of chromosome 5 [17], which has a high GC% (Fig. 1e and 1f). A widespread phenomenon on GC% accumulation near the subtelomeres of eukaryotic chromosomes has been explained by the GC-biased gene conversion, which occurs during DNA repair after meiotic crossing over and which favors ambiguities with G or C nucleotides over A and T [10, 18]. In addition, the same subtelomeric region contains in the eutherian genomes the beta-globin gene cluster, which has contributed to innovations in mammalian oxygen flow during pregnancy [19]. We subsequently looked for landscape synapomorphies in which the eutherian and metatherian lineages maximally diverged at the level of GC%. One of the most outspoken examples of an Eutherian (but not Metatherian) accumulation of GC% is found in the subtelomeric q-arm of human chromosome 14. Indeed, this feature is found in all analyzed Eutherian species (Fig. 1c), but is completely absent in the 4 studied Metatherians Fig. 1c), as well as in other vertebrates [1]. This region contains a gene complex that plays a fundamental role in some of the differences between Eutherian and Metatherian reproductive physiology and adaptive immunity. First, this subtelomeric region contains the DLK1-DIO3 region, which also contains a large cluster of Eutherian miRNA and snoRNA genes [20] that are relevant for placental growth and placental function [21, 22]. Importantly, the locus is imprinted in Eutheria but not in Metatheria [20]. The imprinted DIO3 gene is a key enzyme that regulates the activity of thyroid hormone in target cells, in particular placenta and brain [23]. Placental expression of DIO3 was mentioned as being crucial for protecting the developing fetal brain against maternal thyroid hormone [24]. Interestingly, the DLK1-DIO3 locus has also been implicated in the Eutherian adaptation of newborn pups to extrauterine environment by stimulating the maturation and activity of brown fat cells that generate heat [25]. In this respect, it may be relevant that after birth the Metatherian pups are protected against heat loss by the maternal pouch, whereas the Eutherian young are more exposed to environmental temperature drops.

The subtelomeric q-arm of chromosome 14 not only contains key genes for placental function but it also contains the immunoglobulin heavy chain cluster (IGH), which is one of the pillars of the adaptive immune system in vertebrates [26]. Interestingly, the complexity of the immunoglobulin heavy chain cluster in the eutherian line of mammals is much greater than in Metatheria [27–29]. Some Eutherian lines have evolved specific innovations such as the single chain immunoglobulins in camelids [30]. On the contrary the Metatherian diversity of immunoglobulins is much more dependent on the complexity of the lambda light chain locus (IGLλ) [27, 28] and it is of interest to note that this locus is present in a subtelomeric region with increased GC% in the marsupial genomes (Additional File 4: Fig. S2a). Indeed, studies using FISH probes in *Monodelphis domestica* [31] and in wallaby [32] have localized the IGLλ at a subtelomeric region, which is associated with accelerated evolution in genome landscapes based on the *Monodelphis domestica* gene order, while IGH is situated at an interstitial region of chromosome 1 (Fig. 4a). Conversely, Eutherians such as primates, mouse [33] and dogs have a conserved subtelomeric location of heavy chain locus while the IGLλ is interstitial (Additional File 4: Fig. S2a). This conservation can be extended to other lineages of placental mammals such as alpaca [34], giant panda [35], rabbit [36] and cow [37]. This example from the adaptive immune system illustrates that lineage-specific landscape traits can be correlated to important aspects of physiology other than reproductive biology. The DLK1-DIO3 locus represents an example of an Eutherian genome region with lineage-specific accumulation of GC% and accelerated protein evolution. On the other hand, the subtelomeric q-arm of chromosome 6 in *Monodelphis domestica* (Fig. 1f) contains the HOXA gene cluster, which plays a crucial role in Eutherian uterine development, a process in which the two Müllerian ducts fuse during the female reproductive organogenesis [38]. The seminal role of HOXA13 in this process has been demonstrated both in knockout mouse models [39] and in patients with hand-foot-genital syndrome (OMIM 14,000) [39, 40]. In some families with hand-foot-genital syndrome there is a stable expansion of poly-alanine tracts in the GC-rich first exon of the HOXA13 protein [41–46]. Among the developmental abnormalities that have been reported are didelphic uteri, caused by incomplete fusion of the Müllerian tubes [41]. The lineage-specific differences in the Eutherian/Metatherian HOXA13 sequence, which point in the direction of a unique extra poly-alanine region in the four studied Metatherians (Additional file 4: Fig. S2c) are therefore noteworthy. We believe that further detailed analysis of HOXA13 orthologues and site-directed experimental mutagenesis will be required to better understand the relationship between HOXA13 differences between Eutherians and Metatherians and their effects on uterine anatomy and function.

Finally, our proof-of-concept phylogenetic analysis paves the way for further research into Bayesian model selection and posterior inference under molecular clock models to determine genome-wide landscapes of protein evolution. Such procedures are complex, time-consuming and may ultimately benefit from a more clever modeling exercise where a limited number of analyses attempt to describe larger genomic regions than just a single gene using hierarchical phylogenetic models for example. Therefore, the currently proposed sliding window approach serves as an efficient method to construct genome-wide landscapes to study lineage-specific regional effects on the evolutionary rate of protein divergence.

## Conclusion

A systematic study of divergence of orthologous proteins in mammalian genomes has resulted in landscapes in which lineage-specific regional effects on the molecular clock rates can be visualized. We hypothesize that loci that contain the most pronounced subtelomeric incremental effects on these molecular clock rates are privileged sites for master genes that control important functions such as intra-uterine life and the generation of immunoglobulin diversity.

## Methods

### Retrieving of genome data for a typical vertebrate gene set

Data for GC content (GC%), amino acid usage (amount of glycine, alanine, arginine and proline; GARP%) and protein divergence were downloaded for 4 Metatheria and 12 Eutheria using scripts as previously described [1]. For the Metatheria, we used all four species for which we could retrieve genetic information from NCBI: *Monodelphis domestica, Phascolarctos cinereus, Sarcophilus harrisii* and *Vombatus ursinus*. For the Eutheria, we selected species covering different niches and spanning the three largest clades (4 species per clades): Afrotheria (*Chrysochloris asiatica, Loxodonta africana, Orycteropus afer afer* and *Trichechus manatus latirostris)*, Euarchontoglires (*Callithrix jacchus, Cricetulus griseus*, *Oryctolagus cuniculus,* and *Rattus norvegicus)* and Laurasiatheria (*Equus asinus*, *Felis catus domestica, Orcinus orca* and *Sus scrofa)*. The human genome only served as a reference genome to construct our data matrix, but data of human GC%, GARP% and protein divergence were not used in our analysis. Protein-encoding genes were included in our analysis if the gene was present in both Metatheria and Eutheria to exclude lineage-specific genes, yielding a total set of 15,727 genes. For the genome of the *Monodelphis domestica* (Metatheria), we downloaded the location of the genes to get more information about the characteristics and their physical location. Additionally, for the genome of the pig, *Sus scrofa*, the position of the genes was downloaded to serve a second template for the Eutheria. A list of transcript IDs is provided in Additional file 6: Table S3.

### Sliding window approach, protein divergence and heatmaps

We used the same sliding window as previously described [1]. In short, key metrics (GC%, GARP%, protein divergence) were graphically represented on the genome as a regional average. For every gene on location *L*_*k*_*,* a sliding window metric was defined averaging the metric’s value over 100 neighboring genes at positions *L*_*k−50*_ to *L*_*k*+*50*_. All metrics in the figures are displayed using the sliding window approach.

Protein identity between pairs of orthologous proteins was calculated using EMBOSS Stretcher (BLOSUM62 substitution matrix). The protein identity score was calculated as the number of matching residues divided by the length minus the number of gaps to exclude low protein identities as a consequence of incomplete sequence information. Protein identities below 30% were discarded. Protein divergence was calculated as 1 − protein identity. Divergence times between species were obtained from timetree.org [7].

According to the molecular clock hypothesis, the percentage of changed amino acid residues in an orthologous protein sequence (%protein divergence, abbreviated as PD%) is proportional to the time (t) between the compared species and their split from the last common ancestor [3]. This linear relationship can be written as follows:

$${\text{PD}}\% \, = \,{\text{k}}.{\text{ t}}.$$

The constant k in the equations is known as the evolutionary rate or molecular clock constant [3]. Interestingly, the exact size of k greatly depends on the type of protein that is studied and the mechanism behind this difference is not fully understood. Moreover, for the same type of protein, different values of k were found within different phylogenetic lineages [47]. To study this problem, we first determined the average of all molecular clock constants when comparing the genome-wide set of orthologous proteins of a particular pair of species: PD^av^% = k^av^. t

We were interested in the possibility that the regional position of a gene in a genome landscape could influence k. To assess this possibility, we normalized the protein divergence of all orthologous proteins in a pair of species by dividing PD% by PD^av^.:

$${\text{nPD}}\% \, = {\text{ PD}}\% \, /{\text{PD}}^{{{\text{av}}}} \% = \, {{\left( {{\mathbf{k}} \, . \, {\mathbf{t}}} \right)} \mathord{\left/ {\vphantom {{\left( {{\mathbf{k}} \, . \, {\mathbf{t}}} \right)} {\left( {{\mathbf{k}}^{{{\mathbf{av}}}} . \, {\mathbf{t}}} \right)}}} \right. \kern-\nulldelimiterspace} {\left( {{\mathbf{k}}^{{{\mathbf{av}}}} . \, {\mathbf{t}}} \right)}} = \, {{\mathbf{k}} \mathord{\left/ {\vphantom {{\mathbf{k}} {{\mathbf{k}}^{{{\mathbf{av}}}} }}} \right. \kern-\nulldelimiterspace} {{\mathbf{k}}^{{{\mathbf{av}}}} }}.$$

Such normalization eliminates the influence of time to the common ancestor on the measured result. To investigate a regional contribution of k in genome-wide landscapes we calculated nPD% with sliding window with a centered gene and its 100 neighbors (50 on each side). This calculation was repeated for the 120 possible pairwise comparisons between any two classes of the study. Heatmaps were made in Microsoft Excel with conditional formatting by plotting a table of the sliding window values (for GC or nPD%).

### Circos plots

Circos plots (*Homo sapiens* vs *Sus scrofa* and *Homo sapiens* vs *Monodelphis domestica*) were generated using the synteny portal Syncircos (http://bioinfo.konkuk.ac.kr/synteny_portal/htdocs/synteny_circos.php; settings resolution 150,000 bp). These Circos plots show relationships for all genetic material. We only displayed the lines exceeding the threshold (for human Fig. 3a and for *Monodelphis domestica* Fig. 3b) simplifying the Circos plots. Lines were colored green when the genes were located at the end of the chromosomes in both species. Black lines show that the genes are not located at the ends of the chromosome in both species (if the genes are located at the end of the chromosome in only one species, the line will have a black color).

### Phylogenetic analysis

To investigate the differences in evolutionary rates between Metatherians and Eutherians in the context of their evolutionary history, we performed phylogenetic reconstruction of the sixteen species of interest under four different clock models: a strict clock, a fixed local clock (with one local clock on the Metatherian lineage and another on the Eutherian lineage; [48]), an uncorrelated relaxed clock with an underlying exponential distribution [49], and an uncorrelated relaxed clock with an underlying lognormal distribution. Relaxed clock models are among the most popular models used in phylogenetic inference, owing to their flexibility while not requiring many parameters to be estimated. These models assume that each branch in a phylogenetic tree evolves according to a unique evolutionary rate, drawn from an underlying distribution. We applied a Bayesian model selection approach to determine which clock model best describes each amino acid data set. We considered a subset of genes from each of three chromosomal positions for Bayesian phylogenetic inference and model selection. After filtering out genes for which there were missing data for one or more taxa, we constructed multiple sequence alignments using MUSCLE [50]. This yielded 16 alignments for gene list 1, 63 for gene list 2, and 70 for gene list. For gene lists 2 and 3 we truncate the lists to the 16 top genes for ensuing phylogenetic analysis.

We performed Bayesian phylogenetic reconstruction using BEAST 1.10.5 [51]. For each gene, we undertook phylogenetic reconstruction under the four aforementioned clock models. In addition to enforcing that the Metatherian species form a monophyletic clade we enforced monophyly for Afrotheria, Euarchontoglires, and Laurasiatheria so that a calibration prior could be assigned the most recent common ancestor of each of these clades, in order to obtain a time-calibrated phylogeny. We jointly estimated all parameters of interest, including the topology and branch lengths, using the Markov chain Monte Carlo (MCMC) algorithm as implemented in BEAST. We assumed the default priors as suggested in BEAUti [51] but add calibration priors to a number of internal nodes of the phylogeny. To inform the molecular clock models and be able to estimate divergence times, we included three normally distributed node calibration priors determined by meta-analyses of between-clade divergence times (timetree.org). These calibration priors relate to the Metatheria-Eutheria divergence (μ = 158.5MYA, σ^2^ = 3.85), the divergence of Afrotheria from other eutherians (μ = 104, σ^2^ = 2.55), and the divergence of Euarchontoglires from Laurasiatheria (μ = 96, σ^2^ = 2.55). We performed initial Bayesian model testing using various amino acid substitution models, which revealed that the JTT amino acid model [52] yielded the best relative fit to the data, along with modeling site heterogeneity through a discretized gamma distribution [53]. For each reconstruction, we performed a BEAST analysis for 2,000,000 iterations, which was sufficient for all analyses to converge and to accumulate over 200 effective samples for all inferred parameters, as assessed using Tracer [54]. Parameters were sampled every 2000 iterations after discarding 10% of states as burn-in. For each posterior tree distribution, we constructed a maximum clade credibility tree to summarize phylogenetic results. We performed (log) marginal likelihood estimation using a generalized stepping-stone (GSS; [55]) algorithm to determine which clock model yields the best fit for each amino acid data set. Each power posterior within the GSS approach ran for 100,000 (plus 10% burn-in) iterations for 50 path steps. Parameters were sampled every 500th iteration and (log) marginal likelihoods were calculated for each clock model for each gene. This resulted in a total of 192 analyses to be run as part of the Bayesian model selection exercise to determine the optimal molecular clock model for each amino acid data set and to obtain accurate estimates of all analysis parameters including the tree topology and branch lengths.

## Supplementary Information

**Additional file 1: Figure S1.** Heatmap of GC% in the order of the pig genome. Similar to the heatmap in Fig. 1c, the highest GC% for the Eutheria is often located at the end of the chromosomes, while high GC% for Metatheria is often towards the middle of the chromosomes.

**Additional file 2: Table S1.** Species pairs for heatmaps in Fig. 3. 120 pairs of species can be formed from the 12 Eutheria and 4 Metatheria in our study. Here is a list coupling the number to pair of species.

**Additional file 3: Table S2.** List of genes exceeding the threshold of a fast evolving set in Eutheria and Metatheria. There are 1,833 Eutherian genes above the threshold and 1,504 metatherian genes above the threshold. There are 345 common genes in the two gene sets.

**Additional file 4: Figure S2.** Examples of master gene complexes in subtelomeric regions of the human and *Monodelphis domestica* genome. a) chromosome mapping of IGF2, which is in a subtelomeric GC rich region both in the human and *Monodelphis domestica* genomes. On the contrary, discordance is seen for the HOXA-gene cluster, which is subtelomeric in *Monodelphis domestica* but not in the human genome. Discordance is also seen for the immunoglobulin light chain lambda locus (IGLλ—only subtelomeric in *Monodelphis domestica*) and the heavy chain locus (IGH—subtelomeric in the human genome). In Eutherian genomes IGH is in proximity to DIO3 and a large microRNA gene cluster that regulates placental/fetal interactions. **b)** GC accumulation may also affect regulatory sequence such as large CpG islands that non only overlap with coding information but that also regulate expression of non-coding RNA. **c** Schematic representation of HOXA13 in Eutheria and Metatheria. Some patients with hand-foot-genital syndrome have mutations in the HOXA13 gene that are extensions of the poly-alanine (polyA) tracts. Metatheria have a conserved extra polyA tract (yellow).

**Additional file 5: Figure S3.**Figure S3: Gene density and CpG islands at human chr11p15. The subtelomere of the p-arm of human chromosome 11 contains approximately 200 CpG islands in a gene dense area. The second largest CpG island containing 330 base pairs is located in a bidirectional promotor (Fig. S2b) and covers the first exon of the IGF2 gene (red circle).

**Additional file 6: Table S3.** Transcript ID’s of all 16 used species. This file contains the list of the 15,727 used genes with NM/XM-numbers for all species used in our analysis.

## Data Availability

Data were extracted from publicly available GenBank files. Further datasets generated and/or analyzed during the current study are available from the corresponding author on reasonable request.

## Abbreviations

GC%: GC content
GARP%: % Amino acids glycine (G), alanine (A), arginine (R) and proline (P) in the protein.
nPD%: Normalized protein divergence %
FISH: Fluorescence in situ hybridization
IGH: Immunoglobulin heavy chain
IGLλ: Immunoglobulin light chain
