# Supplementary figures and images for: Regional effect on the molecular clock rate of protein evolution in Eutherian and Metatherian genomes
Source: BMC Ecol Evol. 2021 Aug 4;21:153. doi: 10.1186/s12862-021-01882-x (PMC8336415; doi:10.1186/s12862-021-01882-x)

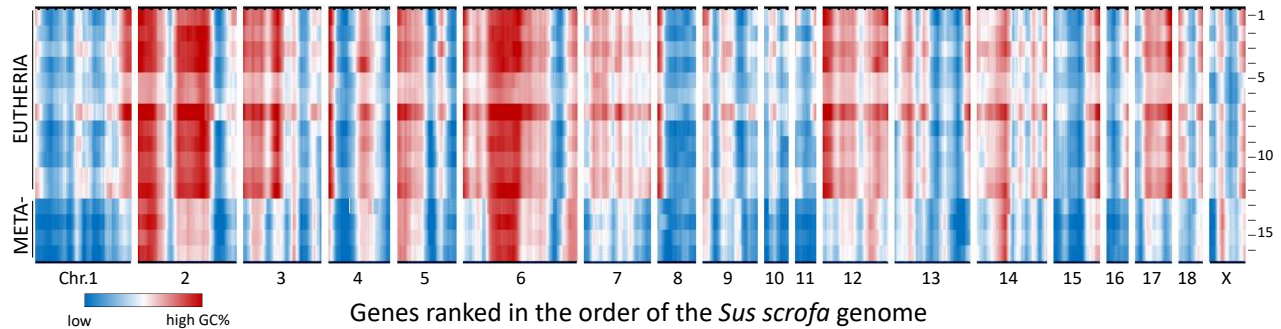

Supplement: Supplementary file 1 — Additional file 1: Figure S1. Heatmap of GC% in the order of the pig genome. Similar to the heatmap in Fig. 1c, the highest GC% for the Eutheria is often located at the end of the chromosomes, while high GC% for Metatheria is often towards the middle of the chromosomes. [file 12862_2021_1882_MOESM1_ESM.pdf]

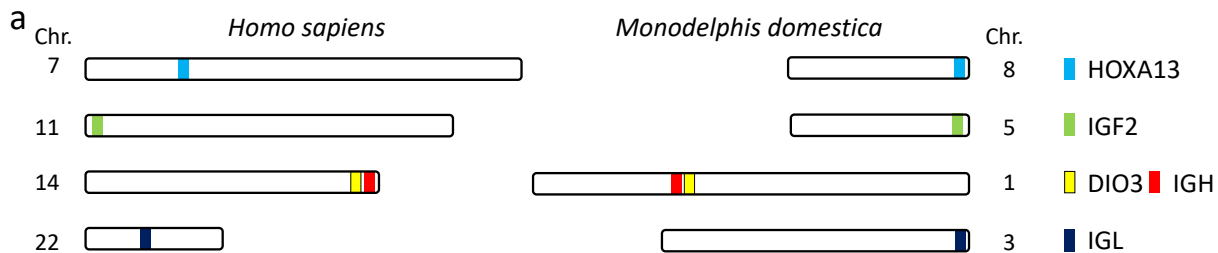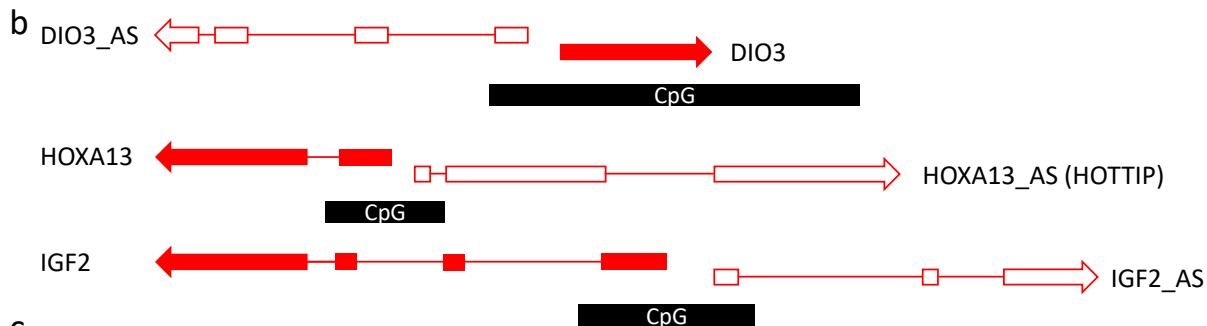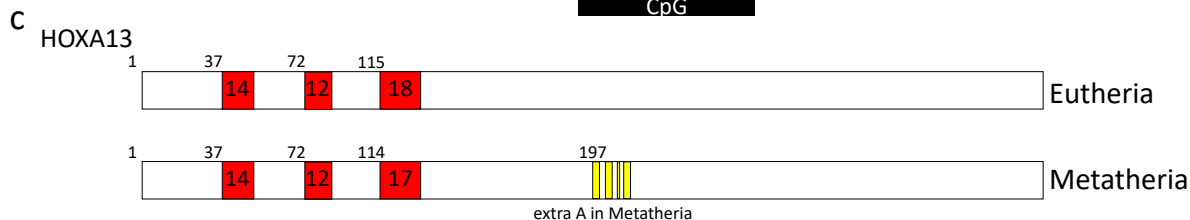

Supplement: Supplementary file 4 — Additional file 4: Figure S2. Examples of master gene complexes in subtelomeric regions of the human and Monodelphis domestica genome. a) chromosome mapping of IGF2, which is in a subtelomeric GC rich region both in the human and Monodelphis domestica genomes. On the contrary, discordance is seen for the HOXA-gene cluster, which is subtelomeric in Monodelphis domestica but not in the human genome. Discordance is also seen for the immunoglobulin light chain lambda locus (IGLλ—only subtelomeric in Monodelphis domestica) and the heavy chain locus (IGH—subtelomeric in the human genome). In Eutherian genomes IGH is in proximity to DIO3 and a large microRNA gene cluster that regulates placental/fetal interactions. b) GC accumulation may also affect regulatory sequence such as large CpG islands that non only overlap with coding information but that also regulate expression of non-coding RNA. c Schematic representation of HOXA13 in Eutheria and Metatheria. Some patients with hand-foot-genital syndrome have mutations in the HOXA13 gene that are extensions of the poly-alanine (polyA) tracts. Metatheria have a conserved extra polyA tract (yellow). [file 12862_2021_1882_MOESM4_ESM.pdf]

chr11:1-2,793,519 2,793,519 bp.

enter position, gene symbol, HGVS or search terms

go

chr11 (p15.5) 11p15.4 15.1 p13 11p12 11.2 13.4 q14.1 q21 22.1 q22.3 q23.3 q25

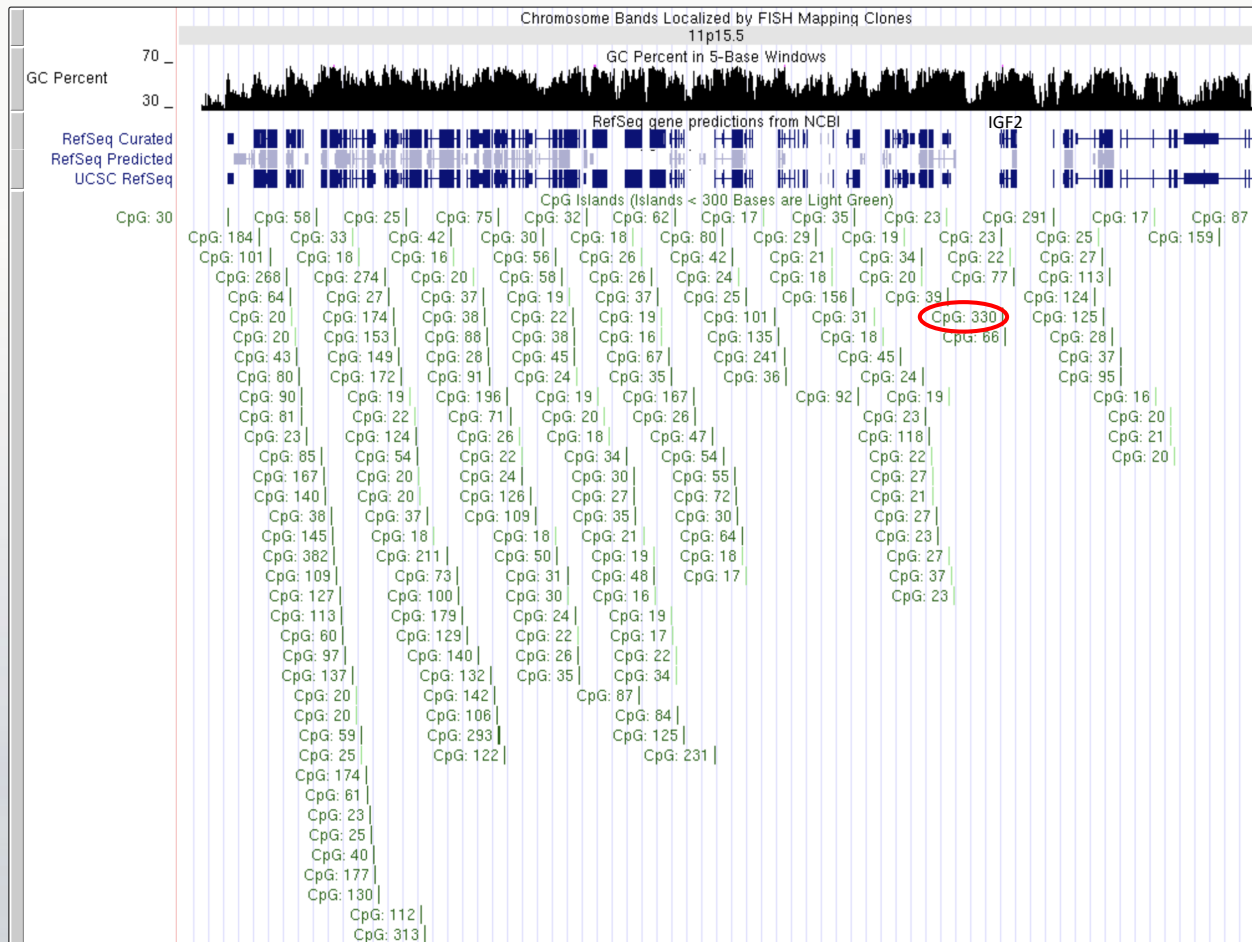

Supplement: Supplementary file 5 — Additional file 5: Figure S3.Figure S3: Gene density and CpG islands at human chr11p15. The subtelomere of the p-arm of human chromosome 11 contains approximately 200 CpG islands in a gene dense area. The second largest CpG island containing 330 base pairs is located in a bidirectional promotor (Fig. S2b) and covers the first exon of the IGF2 gene (red circle). [file 12862_2021_1882_MOESM5_ESM.pdf]
